# *PpNUDX8*, a Peach NUDIX Hydrolase, Plays a Negative Regulator in Response to Drought Stress

**DOI:** 10.3389/fpls.2021.831883

**Published:** 2022-02-16

**Authors:** HuaJie He, YuZheng Zhang, BinBin Wen, XiangGuang Meng, Ning Wang, MingYun Sun, Rui Zhang, XueHui Zhao, QiuPing Tan, Wei Xiao, DongMei Li, XiLing Fu, XiuDe Chen, Ling Li

**Affiliations:** ^1^College of Horticulture Science and Engineering, Shandong Agricultural University, Taian, China; ^2^State Key Laboratory of Crop Biology, Shandong Agricultural University, Taian, China; ^3^Shandong Province Collaborative Innovation Center for High-Quality and High-Efficiency Vegetable Production, Taian, China; ^4^College of Life Sciences, Shandong Agricultural University, Taian, China

**Keywords:** peach, NUDIX hydrolase, drought stress, ABA, NAD/NADH

## Abstract

Drought stress is a serious abiotic stress source that affects the growth and fruit quality of peach trees. However, the molecular mechanism of the NUDIX hydrolase family in peaches in response to drought stress is still unclear. Here, we isolated and identified the *PpNUDX8* (Prupe.5G062300.1) gene from the peach NUDIX hydrolase family, and found that *PpNUDX8* has a typical NUDIX hydrolase domain. In this study, we performed 15% PEG6000 drought treatment on peach seedlings, and qRT–PCR analysis showed that 15% PEG6000 induced the transcription level of *PpNUDX8*. Overexpression of *PpNUDX8* reduced the tolerance of calli to 4% PEG6000 treatment. Compared with wild-type apple calli, *PpNUDX8* transgenic apple calli had a lower fresh weight and higher MDA content. After 15% PEG6000 drought treatment, *PpNUDX8* transgenic tobacco had a greater degree of wilting and shorter primary roots than Under control conditions. The chlorophyll, soluble protein, and proline contents in the transgenic tobacco decreased, and the MDA content and relative conductivity increased. At the same time, *PpNUDX8* negatively regulated ABA signal transduction and reduced the transcriptional expression of stress response genes. In addition, *PpNUDX8* was not sensitive to ABA, overexpression of *PpNUDX8* reduced the expression of the ABA synthesis-related gene *NCED6* and increases the expression of the ABA decomposition-related gene *CYP1* in tobacco, which in turn leads to a decrease in the ABA content in tobacco. In addition, Under control conditions, overexpression of *PpNUDX8* destroyed the homeostasis of NAD and reduced nicotinamide adenine dinucleotide (NADH) in tobacco. After 15% PEG6000 drought treatment, the changes in NAD and NADH in *PpNUDX8* transgenic tobacco were more severe than those in WT tobacco. In addition, *PpNUDX8* also interacted with *PpSnRk1*γ (Prupe.6G323700.1).

## Introduction

Plants live in a constantly changing environment, which leads to abiotic stresses during plant growth and development, and drought and water shortages are abiotic stressors that have a great impact on plants (Guo et al., 2016; Zhao et al., 2019). With the development of global warming, the environment in which plants live is gradually deteriorating, and climate change may increase the frequency of drought stress (Fedoroff et al., 2010). When plants face drought or water shortages, they form corresponding defense mechanisms at the morphological, physiological and molecular levels to protect themselves from drought stress. For example, closing the stomata reduces water loss (Ramachandra Reddy et al., 2004; Hu and Xiong, 2014), by increasing the root to cap ratio and forming more capillary roots to better absorb water (Xiong et al., 2006; Schachtman and Goodger, 2008) and to maintain the osmotic pressure of cells by accumulating proline, soluble sugar, soluble protein, and other osmotic substances (Seki et al., 2007). Improving the activity of a variety of antioxidant enzymes [such as superoxide dismutase (SOD), catalase (CAT), and peroxidase (POD)], to remove the reactive oxygen species (ROS) generated by drought stress (Ramachandra Reddy et al., 2004; Goswami et al., 2013), regulating the synthesis and decomposition pathways of ABA and increasing the content of plant endogenous ABA enhances the drought resistance of plant (Qin and Zeevaart, 2002).

The NUDIX hydrolase family is widely present in various organisms. The members of NUDIX hydrolase usually contain the conserved NUDIX motif GX5EX7REUXEEXGU (where U is a hydrophobic group and X represents any residue)(Bessman et al., 1996; McLennan, 2006). The NUDIX motif is an essential catalytic site for enzymes, and the glutamine residue in its core sequence REUXEE needs to bind to the necessary divalent cations to function. NUDIX hydrolase (NUDX) is a pyrophosphate hydrolase that can hydrolyze nucleoside sugars, dinucleoside polyphosphates, nucleoside triphosphates, and other organic pyrophosphates (Mildvan et al., 2005). These NUDX proteins can be adjusted according to changes in substrate content, by degrading substances that are harmful to plants or accumulating excessive metabolic intermediates, and playing important biological functions in the process of body protection, regulation, and signal transmission (Xu et al., 2004; Ogawa et al., 2005; Yoshimura and Shigeoka, 2015; Ogawa and Yoshimura, 2019).

In recent years, increasing evidence has shown that the NUDX protein plays an important regulatory role in a variety of physiological and biochemical processes, such as cell homeostasis, immune regulation, and abiotic stress response (Xu et al., 2004; Yoshimura and Shigeoka, 2015; Dong and Wang, 2016). Overexpressed *AtNUDX2* maintains NAD + and ATP levels by recovering nucleotides from free ADP-ribose molecules under oxidative stress conditions, thereby increasing tolerance to oxidative stress (Ogawa et al., 2009). Both the *AtNUDX6* and *AtNUDX7* genes are involved in the regulation of the stress response and plant defense. *AtNUDX6*, as a positive regulator of the SA signaling pathway dependent on NPR1 (non-boosting factor of pathogenicity related gene 1), directly participates in plant immune response by regulating the level of reduced nicotinamide adenine dinucleotide (NADH). Knockout (KO) of *AtNUDX6* and the overexpression of *AtNUDX6* showed that the expression of several genes involved in SA-induced NPR1 activation-dependent and TRX-h5 decreased and increased respectively (Ishikawa et al., 2010). The expression of *AtNUDX7* is induced by various types of abiotic stresses, such as drought, salinity, injury and strong light, as well as various oxidation treatments (such as PQ, ozone, O^2–^, and H_2_O_2_). Overexpression of *AtNUDX7* gene and KO (knockout) lead to increased and decreased tolerance to oxidative stress, respectively (Davletova et al., 2005; Jambunathan and Mahalingam, 2006; Ge et al., 2007; Ishikawa et al., 2009, 2010; Jambunathan et al., 2010). According to reports, *KO-NUDX7* plants accumulate high levels of ABA, resulting in a decrease in seed germination rate (Zeng et al., 2014). *AtNUDX7* also plays an indispensable role in seed germination, growth, and development by regulating NAD: NADH homeostasis (Klaus et al., 2005). In grapes, *VvNUDXs* participate in the detoxification process of abiotic and biotic stress, and regulate disease immunity and resistance pathways (Wang et al., 2020).

Although the NUDIX hydrolase family has more been extensively researched on in terms of the abiotic stress response in Arabidopsis, knowledge of the NUDIX hydrolase family involved in the regulation of abiotic stress in peach is still very limited. As one of the most globally important fruits, peach has high nutritional and economic value. Due to the increase in the frequency of extreme weather caused by global warming, abiotic stresses often occur during the growth and development of peaches, resulting in a decline in the yield and quality of peaches. Therefore, understanding the molecular mechanism of the NUDIX hydrolase family in peaches in respond to abiotic stress is of great significance for the further cultivation of new peach varieties with strong adaptability.

Here, we isolated and identified the *PpNUDX8* gene from the peach NUDIX hydrolase family. This study found that *PpNUDX8* played a negative regulatory role in drought stress response. Overexpression of *PpNUDX8* changed the steady state of NAD and NAD/NADH in tobacco and reduced the content of endogenous ABA in tobacco. In addition, we also found that *PpNUDX8* interacts with *PpSnRk1*γ. These findings can provide references for genetic engineering breeding.

## Results

### Bioinformatic Analysis of *PpNUDX8*

**FIGURE 1:**
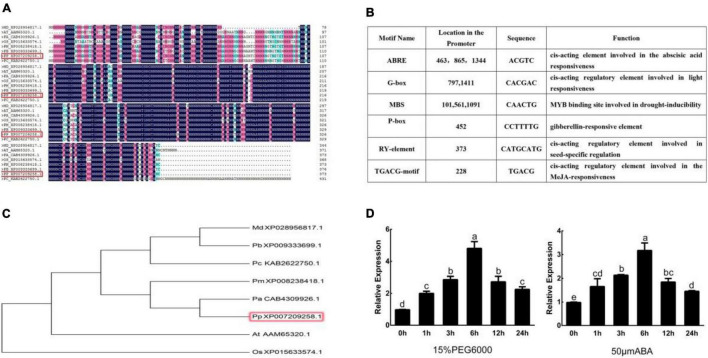
Bioinformatics analysis of *PpNUDX8*. **(A)** Protein multiple sequence alignment of *PpNUDX8*. **(B)** Analysis of cis-elements in the promoter of *PpNUDX8*. **(C)** Phylogenetic tree analysis of *PpNUDX8.* **(D)** Expression profile of *PpNUDX8* in peach seedlings under 15% PEG 6000 and ABA treatment. These error bars represent the mean ± SD of independent biological triplicates. According to the analysis of variance and Duncan’s test, different letters represent significant differences (*P* < 0.05).

*PpNUDX8* was amplified with the cDNA library of peach as a template. The coding sequence (CDS) of *PpNUDX8* is 1122 bp in length, encodes a protein of 373 amino acids, and has a predicted molecular weight of 41.67 kD. The results of protein multiple sequence alignment showed that the *PpNUDX8* protein has a typical NUDIX hydrolase domain (Figure 1A). Phylogenetic tree analysis showed that *PpNUDX8* is similar to apricot and plum (Figure 1C). Analysis of the cis-elements of the *PpNUDX8* promoter showed that *PpNUDX8* has ABA-responsive originals, methyl -jasmonate-responsive originals, gibberellin-responsive originals, drought-induced response originals, and light-responsive originals, etc (Figure 1B). Since *PpNUDX8* contains ABA response elements and drought-induced response elements, we speculated that *PpNUDX8* may be involved in the response to drought stress. Therefore, we performed 15% PEG 6000 and ABA treatments on 4-week-old peach seedlings. Through qRT–PCR analysis, we found that *PpNUDX8* responds to drought and ABA signals (Figure 1D).

### Overexpression of *PpNUDX8* Reduces the Tolerance of Transgenic Apple Calli to Drought

**FIGURE 2:**
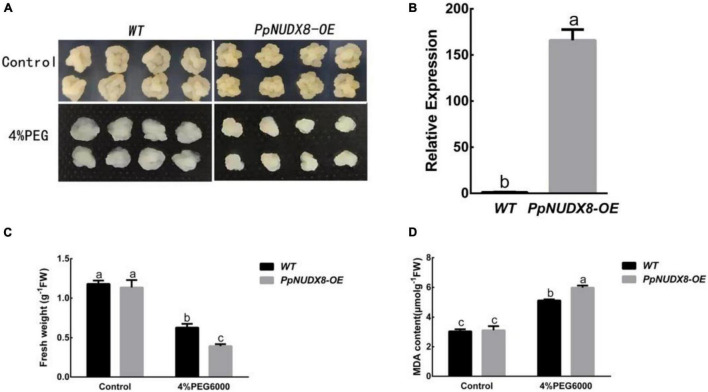
Determination of resistance of PpNUDX8 transgenic apple calli to 4% PEG6000. **(A)** The growth phenotype of WT and transgenic apple calli treated on MS and MS + 4% PEG 6000 medium for 15 days. **(B)** Analysis of *PpNUDX8* expression in WT and *PpNUDX8-OE* transgenic calli by qRT–PCR. **(C)** The fresh weight of apple callus in panel **(A)**. **(D)** The MDA content of apple callus in panel **(A)**. The error bars represent the mean ± SD extracted from independent biological triplicates. According to the analysis of variance and Duncan’s test, different letters represent significant differences (*P* < 0.05).

In the 15% PEG6000 drought treatment of peach seedlings, we found that *PpNUDX8* responds to drought. Therefore, to further study the role of *PpNUDX8* in drought stress, we constructed the overexpression plasmid *PpNUDX8-OE*, which was transferred into Wanglin apple calli through Agrobacterium tumefaciens (LBA4404). Subsequently, through qRT–PCR analysis, we found that the expression of *PpNUDX8* in apple calli was significantly higher than that in wild-type (WT) calli (Figure 2B), indicating that the plasmid *PpNUDX8-OE* was successfully transferred into Wanglin apple calli. In addition, we used 4% PEG6000 to simulate drought treatment of *PpNUDX8* transgenic calli. After 10 days of 4% PEG6000 treatment, we found that the callus growth of *PpNUDX8* transgenic apples was significantly worse than that of WT apples (Figure 2A), and the fresh weight was lower (Figure 2C). Subsequently, we measured the MDA content in the *PpNUDX8* transgenic callus and WT calli and found that the MDA content in the transgenic calli was higher than that in the WT callus (Figure 2D). These date indicate that overexpression of *PpNUDX8* reduces the tolerance of transgenic apple calli to drought.

### Overexpression of *PpNUDX8* Reduces the Drought Tolerance of Transgenic Tobacco

**FIGURE 3:**
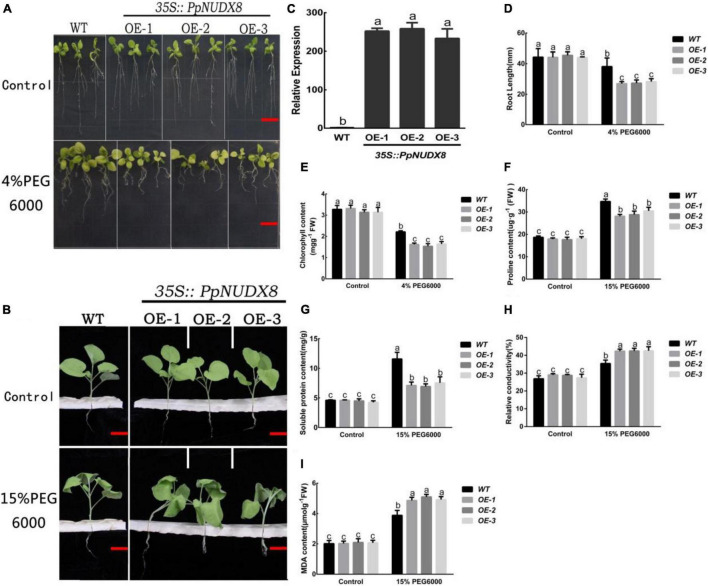
Measurement of drought tolerance of *PpNUDX8* transgenic tobacco and WT. **(A)** The growth phenotype of WT and *PpNUDX8* transgenic tobacco treated on MS and MS + 4% PEG 6000 medium for 10 days. Scale bar = 15 mm. **(B)** The growth phenotype of WT and *PpNUDX8* transgenic tobacco treated with 15% PEG6000 for 4 h. Scale bar = 25 mm. **(C)** Expression analysis of WT and *PpNUDX8*-OX transgenic tobacco lines by RT-qPCR. **(D)** Analysis of the primary root length in panel **(A)**. **(E)** Chlorophyll content in panel **(A)**. **(F–I)** The proline content **(F)**, soluble protein content **(G)**, relative conductivity **(H)**, and MDA content **(I)** in panel **(B)**. The error bars represent the mean ± SD extracted from independent biological triplicates. According to the analysis of variance and Duncan’s test, different letters represent significant differences (*P* < 0.05).

To further study the physiological functions of *PpNUDX8*, we obtained three *PpNUDX8* overexpression transgenic tobacco lines (OE-1, OE-2, and OE-3) through Agrobacterium (LBA3101) transformation. qRT–PCR analysis showed that in these tobacco overexpression lines, the expression level of *PpNUDX8* was significantly higher than that of the WT seedlings (Figure 3C). To study the role of *PpNUDX8* in drought stress, we transferred 4-day-old WT and *PpNUDX8* transgenic tobacco seedlings to MS medium containing 4% PEG6000 for 10 days. As shown in the figure, the growth of WT and transgenic seedlings under the control conditions was basically the same (Figure 3A). After treatment with 4% PEG6000, the growth of *PpNUDX8* transgenic tobacco was significantly lower than that of WT seedlings (Figure 3A), and the primary root length of *PpNUDX8* transgenic tobacco was significantly shorter than that of WT tobacco (Figure 3D). At the same time, the total chlorophyll content of WT leaves was 2.214266 mg/g, while the chlorophyll content of *PpNUDX8* transgenic tobacco was between 1.53 and 1.64 mg/g, which was significantly lower than that of WT seedlings under 4% PEG6000 conditions (Figure 3E). In addition, we used 15% PEG6000 solution to treat 4-week-old WT and *PpNUDX8* transgenic tobacco seedlings that grew consistently. After 4 h of treatment, both WT and transgenic seedlings showed symptoms of wilting and leaf curling, but the degree of wilting of transgenic seedlings was significantly greater than that of WT seedlings (Figure 3B). In addition, we compared the changes in soluble protein, proline, malondialdehyde (MDA) content and relative conductivity of WT and *PpNUDX8* transgenic seedlings under control and 15% PEG6000 conditions. The results showed that Under control conditions, the soluble protein, proline, MDA contents and relative conductivity of WT and *PpNUDX8* transgenic tobacco seedlings were similar. After 15% PEG6000 treatment, the soluble protein, proline, MDA contents and relative conductivity of all plants increased, but proline and the soluble protein contents of WT seedlings were higher than that those of transgenic seedlings (Figures 3F,G). Moreover, the relative conductivity contents and MDA of the WT seedlings were lower than those of the transgenic lines (Figures 3H,I). These date indicate that *PpNUDX8* transgenic tobacco is less drought tolerant than WT tobacco.

### Overexpression of *PpNUDX8* Changed the Steady State of Tobacco Nicotinamide Adenine Dinucleotide and Reduced Nicotinamide Adenine Dinucleotide

Nicotinamide adenine dinucleotide (NAD), as a coenzyme for transferring electrons, participates in many redox reactions in cells. Some studies have shown that NAD plays an important role in the response of plants to environmental signals. The dynamic balance of NAD and NADH is essential for plants to cope with abiotic stress (Hashida et al., 2010; Wu et al., 2018).

**FIGURE 4:**
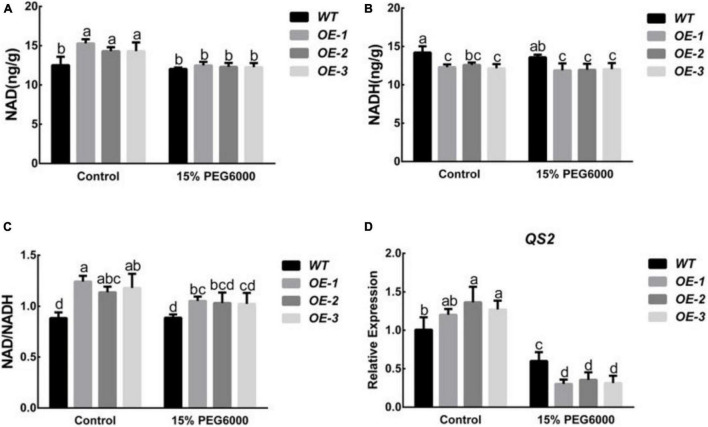
Measurement of NAD and NADH content in *PpNUDX8* transgenic tobacco and WT before and after 15% PEG6000 treatment for 4 h, and analysis of *QS2* expression. **(A)** NAD content. **(B)** NADH content. **(C)** The ratio of NAD and NADH was calculated based on the values shown in panels **(A,B)**. **(D)** Expression analysis of *QS2*. The error bars represent the mean ± SD extracted from independent biological triplicates. According to the analysis of variance and Duncan’s test, different letters represent significant differences (*P* < 0.05).

As a member of the peach NUDIX hydrolase family, *PpNUDX8* may be involved in the homeostasis of NAD in cells. Therefore, we determined the changes in NAD and NADH contents of WT and *PpNUDX8* transgenic tobacco lines before and after 15% PEG6000 treatment. Under control conditions, the NAD content and NAD/NADH ratio of the *PpNUDX8* transgenic line were higher than those of the WT plants, and the NADH content was lower than that of the WT (Figures 4A–C). After 15% PEG6000 treatment, the NAD content, NADH content, and NAD/NADH ratio of the *PpNUDX8* transgenic and WT seedlings decreased, but the NAD content, NADH content, and NAD/NADH ratio of the *PpNUDX8* transgenic lines decreased significantly (Figures 4A–C). At the same time, we determined the expression level of the enzyme *QS*, which plays a key role in NAD biosynthesis. The results showed that under normal circumstances, the expression of *QS2* in the *PpNUDX8* transgenic line was higher than that in the WT line (Figure 4D). After 15% PEG6000 treatment, the *QS2* expression of WT and *PpNUDX8* transgenic lines decreased, but the *QS2* of *PpNUDX8* transgenic lines decreased more than the WT plants (Figure 4D). These date indicate that the overexpression of *PpNUDX8* may change the steady state of NAD and NADH in transgenic tobacco by changing the expression of *QS2*, which plays a key role in NAD biosynthesis. Drastic changes in their steady state may lead to a reduction in the drought tolerance of *PpNUDX8* transgenic tobacco.

### *PpNUDX8* Negatively Regulates Abscisic Acid Signal Transduction and Expression of Stress-Related Genes

**FIGURE 5:**
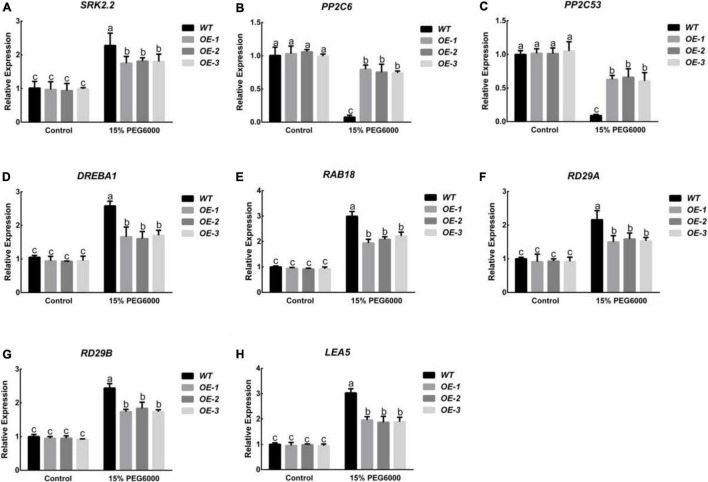
Analysis of the expression levels of *SnRK2.2*, *PP2C6*, *PP2C53*, *DREBA1*, *RAB18*, *RD29A*, *RD29B*, and *LEA5* in *PpNUDX8* transgenic tobacco and WT before and after treatment with 15% PEG6000 for 4 h. **(A)** *SnRK2.2*. **(B)** *PP2C6*. **(C)** *PP2C53*. **(D)** *DREBA1*. **(E)** *RAB18*. **(F)** *RD29A*. **(G)** *RD29B*. **(H)** *LEA5*. The error bars represent the mean ± SD extracted from independent biological triplicates. According to the analysis of variance and Duncan’s test, different letters represent significant differences (*P* < 0.05).

Research on ABA signal transduction and the mechanism of action has always been an important subject in plant stress biology research. In order to further study the role of *PpNUDX8* in drought stress, we detected the transcription levels of several ABA signaling pathway-related genes (*SRK2.2*, *PP2C6*, and *PP2C53*) and stress-related genes (*DREBA1, RAB18, RD29A, RD29B*, and *LEA5*) in tobacco by RT-qPCR. Under control conditions, the expression levels of tobacco genes *SRK2.2*, *PP2C6*, and *PP2C53* in WT and *PpNUDX8* transgenic lines were slightly different, but after 15% PEG6000 treatment, the transcription level of *SRK2.2* in WT was significantly higher In the *PpNUDX8* transgenic lines (Figure 5A). The transcription levels of WT and *PpNUDX8* transgenic lines *PP2C6* and *PP2C53* were reduced, but the transcription levels of *PpNUDX8* transgenic lines *PP2C6* and *PP2C53* were still higher than that of WT (Figures 5B,C). In addition, under control conditions, there was only a slight difference in the transcription levels of stress-related genes (*DREBA1, RAB18, RD29A, RD29B*, and *LEA5*) between the *PpNUDX8* transgenic lines and WT. After 15% PEG6000 treatment, the transcription level of stress-related genes (*DREBA1, RAB18, RD29A, RD29B*, and *LEA5*) in WT was significantly higher than that of *PpNUDX8* transgenic lines (Figures 5D–H). In conclusion, these data indicate that overexpression of *PpNUDX8* may reduce the resistance of tobacco to drought stress by negatively regulating ABA signal transduction and expression of stress-related genes.

### Overexpression of *PpNUDX8* Reduces the Sensitivity of Tobacco to Abscisic Acid

**FIGURE 6:**
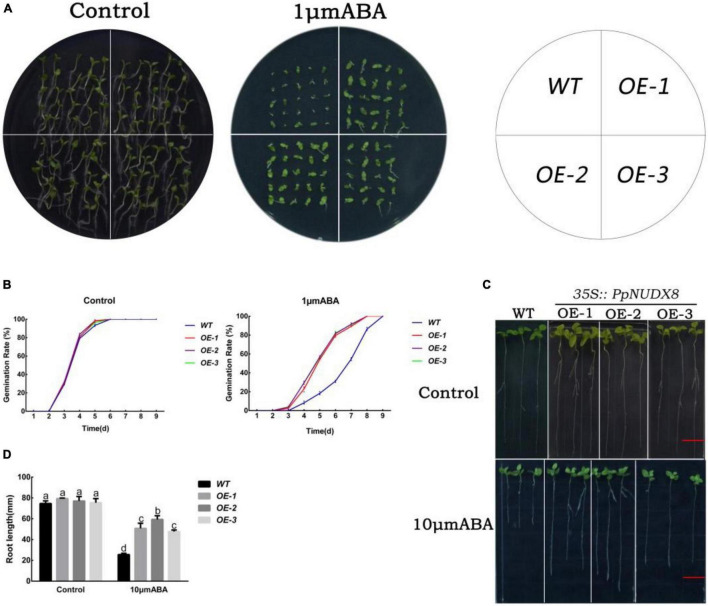
Analysis of abscisic acid (ABA) sensitivity of *PpNUDX8* transgenic tobacco. **(A)** Phenotypes of WT and *PpNUDX8* transgenic lines treated with 0 or 1 μm/L ABA during seed germination. **(B)** Seed germination rate in panel **(A)**. **(C)** Root growth phenotypes of WT and *PpNUDX8* transgenic lines treated with 0 or 10 μm/L ABA in the seedling growth stage. Scale bar = 15 mm. **(D)** The primary root growth in panel **(C)**. The error bars represent the mean ± SD extracted from independent biological triplicates. According to the analysis of variance and Duncan’s test, different letters represent significant differences (*P* < 0.05).

To determine the effect of *PpNUDX8* under ABA treatment, we tested the sensitivity of *PpNUDX8* transgenic tobacco lines under abscisic acid (ABA) treatment. To determine the effect of *PpNUDX8* under ABA treatment, we tested the sensitivity of *PpNUDX8* transgenic tobacco lines under ABA treatment. In the MS plate seed germination test, the germination rates of WT and *PpNUDX8* transgenic tobacco seeds on normal MS medium were similar (Figure 6B). After 1 μm/L ABA treatment, the germination rate of *PpNUDX8* transgenic tobacco seeds was higher than that of WT seeds (Figure 6B). In addition, ABA also significantly inhibited the early growth of WT and *PpNUDX8* transgenic seedlings, but the inhibition of WT seedlings was more serious. We found that the percentage of green cotyledons of *PpNUDX8* transgenic tobacco seedlings was much higher than that of WT seedlings (Figure 6A). In addition, we further tested the sensitivity of WT and *PpNUDX8* transgenic tobacco lines to ABA at the seedling growth stage after germination. After transferring 4-day-old tobacco seedlings to MS medium containing 10 μm/L ABA for 15 days, the *PpNUDX8* transgenic line had longer primary roots than the WT (Figures 6C,D). These date indicate that overexpression of *PpNUDX8* may reduce the sensitivity of tobacco to ABA.

### Overexpression of *PpNUDX8* Reduces the Abscisic Acid Content of Tobacco

**FIGURE 7:**
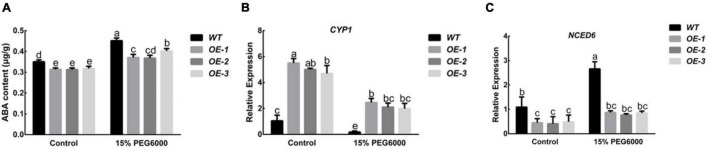
Determination of ABA content in *PpNUDX8* transgenic tobacco before and after 15% PEG6000 drought treatment and expression analysis of *NCED6* and *CYP1*. **(A)** The content of ABA. **(B)** *CYP1*. **(C)** *NCED6*. The error bars represent the mean ± SD extracted from independent biological triplicates. According to the analysis of variance and Duncan’s test, different letters represent significant differences (*P* < 0.05).

Studies have shown that ABA can enhance the drought resistance of plants. After the plants are subjected to drought stress, their endogenous ABA content will increase significantly. Under control conditions, the ABA content of the *PpNUDX8* transgenic line was lower than that of the WT. After 15% PEG6000 for 4 h, the ABA content in all plants increased, but the increase in the *PpNUDX8* transgenic lines was lower than that in the WT seedlings (Figure 7A). In addition, since *NCED* and *CYP* are enzymes that play key roles in the pathways of ABA synthesis and decomposition, respectively, we measured their transcription levels before and after 15% PEG6000 treatment. The results showed that Under control conditions, the expression level of the *CYP1* gene of the *PpNUDX8* transgenic line was much higher than that of WT tobacco, and the expression level of the *NCED6* gene was lower than that of WT tobacco. After 15% PEG6000 treatment, the expression of *CYP1* in both WT and *PpNUDX8* transgenic lines decreased, but the gene expression in *PpNUDX8* transgenic lines was still higher than that of WT (Figure 7B). *NCED6* gene expression increased, but the *NCED6* gene expression of the *PpNUDX8* transgenic line was still much lower than that of WT tobacco (Figure 7C). These date indicate that overexpression of *PpNUDX8* may reduce the ABA content in tobacco by changing the expression of *NCED6* and *CYP1.*

### *PpNUDX8* Interacts With *PpSnRk1*γ

**FIGURE 8:**
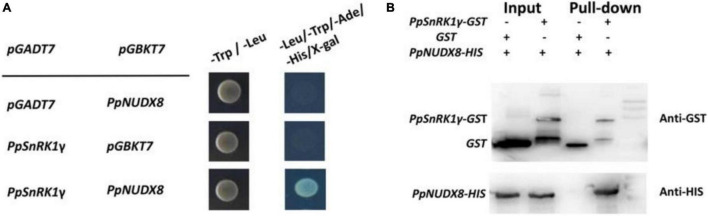
Interaction between *PpNUDX8* and *PpSnRk1*γ. **(A)** Y2H test shows that *PpNUDX8* and *PpSnRk1*γ interact in yeast. **(B)** The interaction between *PpNUDX8* and *PpSnRk1*γ protein in a pull-down assay.

To further explore the biochemical functions of *PpNUDX8* in peaches, we used *PpNUDX8-pGBKT7* as the “bait” to screen the interacting proteins in the peach cDNA library. After multiple transformations of *PpNUDX8*-pGBKT7 and library plasmids, we screened the candidate gene *PpSnRk1*γ. To confirm the interaction between *PpNUDX8* and *PpSnRk1*γ, yeast two-hybrid (Y2H) assays were performed. The full-length cDNA of *PpNUDX8* was inserted into the yeast vector pGBKT7 and the full-length cDNA of *PpSnRk1*γ was inserted into the yeast vector pGADT7. The results showed that *PpNUDX8* interacted with *PpSnRk1*γin yeast (Figure 8A).

To further verify the accuracy of the interaction between *PpNUDX8* and *PpSnRk1*γ, we performed a pull-down experiment. *PpNUDX8*-His and *PpSnrk1*γ-GST fusion proteins were prepared. As shown in Figure 8B, *PpSnrk1*γ was found to be pulled down by *PpNUDX8*, indicating that *PpSnrk1*γ interacts with *PpNUDX8*.

## Materials and Methods

### Plant Materials and Treatments

In the drought and ABA treatment of peach seedlings, 4 weeks old peach seedlings were soaked in a 15% PEG6000 solution and sprayed with 50 μmol/L ABA on peach seedlings, and at 0, 1, 3, 6, and 12 h, the leaves of peach seedlings were collected. WT and transgenic tobacco seeds were sterilized with 4% sodium hypochloride for 10 min, and sown on MS medium containing 3% sucrose and 0.7% agar. After vernalization at 4°C for 4 days, the MS medium plate was transferred to a 24°C light incubator with a 16/8 h light/dark schedule, and a relative humidity of 70%. In the drought treatment apple callus experiment, 15-day-old apple calli were crushed and transferred to MS medium containing 4% PEG6000, and the phenotype was observed and recorded after 10 days of treatment. The relative electrical conductivity and MDA contents of apple calli before and after treatment were measured. For the seed germination test, tobacco seeds were germinated on MS medium supplemented with 0 or 1 μmol/L ABA, and the germination rate was calculated for 10 consecutive days after sowing. One hundred seeds per plant were used to calculate the germination rate and green cotyledons. For the determination of tobacco root length, 4-day-old tobacco seedlings of similar size were vertically cultured in MS medium containing 10 μmol/LABA for another 10 days, and then the primary root length was measured. For MS plate drought treatment, 4-day-old tobacco seedlings were transferred to MS medium containing 4% PEG6000 and cultured for 15 days. In the 15% PEG6000 simulated drought treatment, 3-week-old tobacco seedlings were immersed in a 15% PEG6000 solution, and samples were taken at 0 and 4 h to measure relative conductivity and the contents of MDA, proline, and soluble protein.

### Vector Construction and Genetic Transformation

The full-length CDS of the peach gene *PpNUDX8* was obtained on the Phytozome website^1^. Using the cDNA of peach as a template, the full length *PpNUDX8* gene was cloned, inserted it into the required vector, and DNAMAN software was used for sequence alignment. The primers used for vector construction are listed in Supplementary Table 1. Subsequently, the obtained vector 35S:*PpNUDX8* was transformed into Agrobacterium tumefaciens strains GV4404 and GV3101, and Agrobacterium infection was used to transform Wanglin calli and *Nicotiana tabacum* (Xie et al., 2012). T0 seeds were sown on MS medium containing kanamycin (50 mg/L) for initial selection, and 4 weeks after the seedlings were moved into the plugs, DNA was extracted for transgenic selection. Three homozygous T3 transgenic lines (OE-1, OE-2, and OE-3) were selected for the subsequent experiment.

### RNA Extraction and qRT–PCR

According to the manufacturer’s instructions, a RNA plant extraction kit (Chinese Tiangen) was used to extract the total RNA from apple calli and tobacco and peach seedlings. Reverse transcription was performed with HiScript QRT SuperMix (Vazyme, Nanjing, China). *MdACTIN* (apple), *NtACTIN* (tobacco), and *PpACTIN* (peach) was used as internal controls to complete the qRT–PCR analysis. Each sample was subjected to independent biological triplicate analysis. The primers used for qRT–PCR are listed in Supplementary Table 1.

### Y2H and Pull-Down Experiments

The full-length CDS fragments of *PpNUDX8*, and *PpSnrk1*γ were inserted into pGBKT7 or pGADT7 to generate the *PpNUDX8*-PpGBKT7, and *PpSnrk1*γ-pGADT7 plasmids. Then, transfer the different combinations of pGBD and PGAD were transferred into yeast strains (Y2H) and spread on to SD/-Trp/-Leu and SD/-Trp/-Leu/-Ade-/-His/X-gal selection media at 28°C for 3 days.

The *PpNUDX8* clone was connected to the PET32a vector with a HIS tag, *PpSnRk1*γwas connected to the PGEX4T-1 vector with GST tag, and the recombinant vectors *PpNUDX8*-HIS, *PpSnRk1*γ-GST, and empty PGEX4T-1 were transferred into the BL21 protein expression strain. Then, prokaryotic expression was used to induce the fusion protein and the corresponding kit was used to purify the protein. The purified proteins *PpNUDX8*-HIS and *PpSnRk1*γ-GST were mixed and incubated, and a control (*PpNUDX8*-HIS and empty PGEX4T-1 mixed incubation) was set at the same time. After incubation, part of the mixed solution Input was collected and applied it to the GST column, and the effluent was collected subjected to pull-down experiments after elution.

### Abscisic Acid Content Determination

High performance liquid chromatography-tandem mass spectrometry (HPLC–MS/MS) was used to determine the ABA content of tobacco leaves. Extraction method: Approximately, 0.1 g of tobacco leaf sample was weighed, 1 mL of pre-cooled reagent was added, and the sample was extracted overnight at 4°C prior to centrifugation at 8000 g for 10 min, and the residue was extracted with 0.5 mL of reagent for 2 h. The supernatant was removed after centrifugation and the process was repeated. The two supernatants were combined and evaporated under nitrogen at 40°C until the organic phase was removed. Then, 0.5 mL of reagent was added for the second extraction and decolorization three times, and the upper ether phase was discarded. Reagent three was added to adjust the pH to 2.8. Reagent four was used for three extraction cycles, and the organic phases were combined and evaporated to dryness under nitrogen. A total of 0.5 mL of reagent five was added to the residue and vortexed and shaken to achieve dissolution. After passing the solution through a syringe filter, the sample was analyzed (reagent 1: 80:20:1 methanol/water/acetic acid; reagent 2: petroleum ether; reagent 3: saturated citric acid solution; reagent 4; ethyl acetate; and reagent 5: methanol).

### Physiological Measurement

Total chlorophyll (a + b) measurement (Qin and Zeevaart, 2002). Tobacco leaves and soak were soaked in 95% ethanol under dark conditions for 24 h (during this period, the solution was shaken once every 8 h). Finally, a spectrophotometer was used to determine the total chlorophyll concentration at wavelengths of 665, 646, and 470 nm. Relative conductivity measurement (Lurie and Ben-Arie, 1983). First, the tobacco leaves were rinsed with distilled water then cut into small pieces, and 0.5 g of leaves was put in distilled water. The air was pumped three times, placed at room temperature for 3∼4 h, and the conductivity S1 was measured with a conductivity meter. The sample was boiled for 20 min and cooled to room temperature, and the electrolyte conductivities S2 and S0 were measured. Relative conductivity (%) = 100 × (S1-S0)/(S2-S0). MDA content determination (Ma et al., 2017). The thiobarbituric acid method was used to detect the MDA content. The soluble protein content was determined by the Coomassie Brilliant Blue method.

### Determination of Nicotinamide Adenine Dinucleotide and Reduced Nicotinamide Adenine Dinucleotide Enzyme Activity

According to the instructions of the NAD/NADH detection kit (Shanghai Fanyin Biological), the double antibody sandwich method enzyme-linked immunosorbent assay (ELISA) method was used for NAD and NADH enzyme activity determination.

### Statistical Analysis

SPSS software version 19.0 (SPSS Corp, Chicago, IL, United States) was used for statistical analysis. Duncan’s multiple range test was performed for significant difference analysis with p < 0.05 designated as a significant difference.

## Discussion

Drought stress causes great damage to the growth and development of plants and may lead to premature death of plants in severe cases (Tiwari et al., 2010). Therefore, studying the molecular mechanism of drought stress and the identification of stress response genes should be the focus of plant genetic engineering breeding (Liu et al., 2016; Tan et al., 2016; He et al., 2017). NUDIX hydrolase is a kind of pyrophosphate hydrolase whose hydrolysis substrate mainly includes some coenzymes [NAD(P)H, FAD and coenzyme A] and nucleotide sugars. These compounds are not only metabolic intermediates and coenzymes but also contain some toxic organic compounds, so they must be properly regulated. In addition to participating in the removal of harmful substances and metabolic regulation, NUDIX hydrolase is also closely related to the response of plants to biotic and abiotic stress (Ogawa et al., 2005, 2008; Munoz et al., 2006; Xu et al., 2006; Yoshimura and Shigeoka, 2015). For example, in Arabidopsis, knocking out *AtNUDX6* and overexpressing *AtNUDX6* showed that the expression of several genes involved in SA-induced NPR1 activation-dependent and TRX-h5 decreased and increased, respectively (Ishikawa et al., 2010). The expression of *AtNUDX7* is induced by various types of abiotic stresses, such as drought, salinity, injury and strong light, as well as various oxidation treatments (such as PQ, ozone, O^2–^, and H_2_O_2_). Overexpression of the *AtNUDX7* gene and *KO* lead to increased and decreased tolerance to oxidative stress, respectively (Davletova et al., 2005; Jambunathan and Mahalingam, 2006; Ge et al., 2007; Ishikawa et al., 2009, 2010; Jambunathan et al., 2010).

In this study, the NUDIX hydrolase *PpNUDX8* was isolated and identified from peaches. After 4% PEG6000 drought treatment of *PpNUDX8* transgenic apple calli and tobacco seedlings, it was found that overexpression of *PpNUDX8* reduced the drought tolerance of transgenic apple calli and tobacco (Figures 2A, 3A,B). This result indicates that *PpNUDX8*, as a negative regulator, participates in the tolerance of plants in response to drought stress.

Plants have evolved a variety of mechanisms to resist drought stress (Miller et al., 2010), such as the root system of plants. Some plants have longer root systems under drought stress, which can absorb deep soil water (Hu and Xiong, 2014). The root system is an important organ for plants to absorb water (Wang et al., 2003). Good development of the root system is conducive to the absorption and utilization of water in the soil, thereby enhancing the resistance of plants to drought stress (Wang et al., 2017). In this study, the primary roots of *PpNUDX8* transgenic tobacco after 4% PEG6000 treatment were significantly shorter than those of WT tobacco (Figure 3D), which would not be conducive to improving the water absorption capacity of transgenic tobacco. In addition, under drought stress conditions, the membrane structure related to photosynthesis will be destroyed, which may lead to a decrease in chlorophyll content. According to reports, as the soil moisture content decreases, the chlorophyll content will decrease significantly (Kocheva et al., 2004; Li et al., 2006; Guo et al., 2007). Therefore, maintaining a high chlorophyll content can make more efficient use of light energy, thereby improving the drought resistance of plants. In this study, the chlorophyll content of *PpNUDX8* transgenic tobacco after 4% PEG6000 treatment was lower than that of WT (Figure 3E), which would reduce the photosynthetic capacity of the transgenic tobacco and reduce the drought tolerance of the plant. Plants undergo osmotic adjustment after being subjected to drought stress. Plants accumulate a variety of organic and inorganic substances to increase their concentration in cells, reduce osmotic potential, and improve the water retention capacity of cells under drought stress (Bartels and Sunkar, 2005). Proline and soluble protein act as osmotic regulators to maintain the osmotic balance of cells (Hong-Bo et al., 2005; Ashraf and Foolad, 2007; Maurel et al., 2008). In this study, the proline and soluble protein contents of the *PpNUDX8* transgenic line under 15% PEG6000 treatment were lower than those of WT tobacco (Figures 3F,G), which indicated that the osmotic adjustment ability of *PpNUDX8* transgenic tobacco under 15% PEG6000 treatment was not as good as that of WT tobacco.

Therefore, *PpNUDX8* may be involved in osmotic adjustment. Under stress conditions, the transcription levels of many stress-related genes will be induced (Yamaguchi-Shinozaki and Shinozaki, 2006). *DREBA1* participates in dehydration stress response through ABA-independent signaling pathway (Umezawa et al., 2006). In this experiment, after 15% PEG6000 treatment, the transcription level of DREBA1 in the *PpNUDX8* transgenic line was significantly lower than that of WT (Figure 5D). This means that *PpNUDX8* transgenic tobacco reduced drought tolerance through ABA-dependent and ABA-independent signaling pathways. Advanced Embryogenesis Abundant Protein (*LEA*) can protect biological macromolecules, redirect the distribution of water in cells, combine with inorganic ions, and avoid damage caused by the accumulation of high concentrations of ions under drought stress conditions (Close, 2010). After 15% PEG6000 treatment, the transcription level of *LEA5* in the *PpNUDX8* transgenic line was significantly lower than that of WT (Figure 5H). In addition, the *RAB18*, *RD29A* and *RD29B* genes are very sensitive to various abiotic stressors and are generally involved in drought stress responses (Msanne et al., 2011; Li et al., 2020). After 15% PEG6000 treatment, the expression levels of *PpNUDX8* transgenic lines *RAB18*, *RD29A*, and *RD29B* were significantly lower than WT (Figures 5E–G). These data indicate that *PpNUDX8* reduces drought resistance by regulating the expression level of stress-related genes.

In addition, plants produce a large amount of ROS after being subjected to adverse stress, which aggravates the degree of membrane lipid peroxidation, produces MDA, destroys the integrity of cell membranes, and leads to an increase in electrical conductivity. MDA content and relative conductivity can reflect the degree of damage to the integrity of cell membranes (Hodges et al., 1999; Ozgur and Gevrek, 2008). Therefore, to explore the degree of damage to the cell membrane of *PpNUDX8* transgenic tobacco under 15% PEG6000 treatment, we measured the MDA content and relative conductivity of the transgenic tobacco. The MDA content and relative conductivity of *PpNUDX8* transgenic tobacco were significantly higher than those of WT tobacco (Figures 3H,I). This result indicates that the cell membrane of transgenic tobacco is more severely damaged than WT tobacco under 15% PEG6000 treatment, and the resistance of *PpNUDX8* transgenic tobacco to oxidative stress is weaker than that of WT tobacco (Figure 3B).

Abscisic acid, as a plant hormone that has been extensively studied, can not only regulate seed dormancy and germination, but also respond to abiotic stresses such as drought and water shortage (Kermode, 2005; Zhu, 2016; Nonogaki, 2017). In addition, drought stress can trigger ABA-dependent signaling pathways (Zhu, 2002). According to reports, some NUDIX hydrolases may be involved in the ABA signaling pathway. For example, *KO-AtNUDX7* accumulates high levels of ABA, resulting in a decrease in the seed germination rate (Zeng et al., 2014). In this experiment, after ABA plate treatment on *PpNUDX8* transgenic tobacco seeds, we found that *PpNUDX8* transgenic tobacco was very insensitive to ABA, and the percentage of green cotyledons of *PpNUDX8* transgenic tobacco seedlings was much higher than that of WT seedlings (Figure 6A), and *PpNUDX8* Compared with WT seedlings, the transgenic seedlings had longer primary roots (Figures 6C,D). This observation indicates that *PpNUDX8* may be involved in the ABA signaling pathway. Therefore, we tested the expression levels of *PP2Cs* and *SnRK2s*, the core components of ABA signal transduction. In the ABA signal transduction pathway, *PP2Cs* play a negative regulatory role in ABA signal transduction (Ma et al., 2009; Park et al., 2009). SnRK2s positively regulate the ABA signal transduction pathway, and *SnRK2s* can improve plant tolerance to abiotic stress (Huai et al., 2008; Mao et al., 2010; Zhang et al., 2011). After 15% PEG6000 treatment, the expression level of *SnRK2.2* in the *PpNUDX8* transgenic seedlings was lower than that in the WT seedlings (Figure 5A), while the transcription levels of *PP2C6* and *PP2C53* were higher than those in the WT seedlings (Figures 5B,C). This result indicates that *PpNUDX8* inhibited ABA signal transduction. According to reports, under drought stress, ABA content in plants will quickly accumulate to resist drought stress (Fujita et al., 2011). In this experiment, we measured the ABA content of the *PpNUDX8* transgenic and WT seedlings and found that the ABA content of the *PpNUDX8* transgenic seedlings was lower than that of the WT seedlings before and after drought treatment (Figure 7A). *NCED* and *CYP* are enzymes that play key roles in ABA synthesis and decomposition pathways, respectively. Therefore, we analyzed the transcription levels of *NCED6* and *CYP1* by qRT–PCR. The results showed that the expression of *CYP1* in *PpNUDX8* transgenic tobacco was much higher than that of WT, while the expression of *PpNUDX8* transgenic tobacco *NCED6* was lower than that of WT tobacco (Figures 7B,C). These results indicate that overexpression of *PpNUDX8* may affect the transcription levels of *NCED6* and *CYP1* in transgenic tobacco, leading to a lower ABA content in *PpNUDX8* transgenic lines than in WT plants, which in turn leads to drought tolerance in transgenic plants.

Nicotinamide adenine dinucleotide and NADH are key metabolic intermediates between redox reactions and energy metabolism. The destruction of the dynamic balance of NAD and NADH is likely to affect the accumulation of reactive oxygen species, plant growth, and adaptation to unfavorable environmental conditions (Hashida et al., 2009; Chini et al., 2017; Bockwoldt et al., 2019). For example, deletion of the *AtNUDX7* gene disrupts NADH homeostasis, and *KO-AtNUDX7* accumulates high levels of ABA, resulting in a decrease in seed germination (Zeng et al., 2014). In addition, the NADH content decreased and increased in Arabidopsis overexpressing *AtNUDX6* and knocking out *AtNUDX6*, respectively (Ishikawa et al., 2010). In this experiment, overexpression of *PpNUDX8* affected changes in tobacco NAD, NADH content and NAD biosynthetic enzyme *QS2* expression, both of which were higher than in WT tobacco (Figures 4A,B,D). After 15% PEG 6000 treatment, the NAD and NADH ratios of WT and *PpNUDX8* transgenic lines decreased, but that in *PpNUDX8* transgenic tobacco changed to a greater degree (Figures 4A,B).

In short, these findings can give us a deeper understanding of the regulatory mechanism of *PpNUDX8* under drought stress. This research is helpful to further understand the role of NUDIX hydrolase family in drought stress response, and is expected to be applied to drought improvement in horticultural crop breeding.

## Data Availability Statement

The original contributions presented in the study are included in the article/Supplementary Material, further inquiries can be directed to the corresponding authors.

## Author Contributions

HH, XC, and LL conceived and designed the research. All authors contributed to the article and approved the submitted version.

## Conflict of Interest

The authors declare that the research was conducted in the absence of any commercial or financial relationships that could be construed as a potential conflict of interest.

## Publisher’s Note

All claims expressed in this article are solely those of the authors and do not necessarily represent those of their affiliated organizations, or those of the publisher, the editors and the reviewers. Any product that may be evaluated in this article, or claim that may be made by its manufacturer, is not guaranteed or endorsed by the publisher.

## Footnotes

1
https://phytozome.jgi.doe.gov
