# Supplementary material for: PpNUDX8, a Peach NUDIX Hydrolase, Plays a Negative Regulator in Response to Drought Stress
Source: Front Plant Sci. 2022 Feb 16;12:831883. doi: 10.3389/fpls.2021.831883 (PMC8888663; doi:10.3389/fpls.2021.831883)
Supplement: Supplementary file 1 [file Table_1.pdf]

***PpNUDX8*, a peach NUDIX hydrolase, plays a negative regulator in response to drought stress**

Table S1. Primers used for RT-PCR in this study

**Gene**	**Primers**
MdActin	**5’-TGACCGAATGAGCAAGGAAATTACT-3’**
	**5’-TACTCAGCTTTGGCAATCCACATC-3’**
NtQS2	**5’-CAGCTTATGAGGCAGGACGA-3’**
	**5’-AAGCGGAGCTTGATCGTTGA-3’**
NtNCED6	**5’- ACCCTCTTCAGAAACTTGCATCT-3’**
	**5’-ACCTCAAGCCCATGCTGAAC-3’**
NtSRK2.2	**5’-CCCTGCACCAAGGCTAAAGA-3’**
	**5’-GCTCCTCCGGGTCTTCAAAA-3’**
NtCYP1	**5’-TCAAGTCCTGAAGCAGCTAAAC-3’**
	**5’-ATTCGAGCGTAGTTTTGGCG-3’**
NtPP2C53	**5’-ATGGCCATGGTGGTTCTCAG-3’**
	**5’-GGCTGTGGAGCCTACAGTTT-3’**
NtPP2C6	**5’-ATGGGCATCGTGTTTTTGGTG-3’**
	**5’-CTTGGCCCCTTTCCAGGGTA-3’**
NtNUDX8	**5’-TGCCCCTGGATGAGTTTGTG-3’**
	**5’-TCCAGTGGGAGAGAGATGCG-3’**
NtActin	**5’-GAAGAAGGTCCCAAGGGTTC-3’**
	**5’-TCTCCCTTTAACACCAACGG-3’**
PpNUDX8	**5’-TGCCCCTGGATGAGTTTGTG-3’**
	**5’-TCCTAGCCGGGCGATACATA-3’**
PpActin	**5’-GTTATTCTTCATCGGCGTCTTCG-3’**
	**5’-CTTCACCATTCCAGTTCCATTGTC-3’**
NtDREBA1	**5’- TCAATTTCGCTGACTCGGCT-3’**
	**5’-TCCGCCGTGTAAATAGCCTC-3’**
NtRD29A	**5’- TGCAATATTCACTGGTCAGATGC-3’**
	**5’-GGTGGCCTTGCAAATTCGTC-3’**
Nt RD29B	**5’-GTAGAAGATGAAGGGGCGCA -3’**
	**5’-TTCTGTACCGCCAACTTCCC-3’**
Nt RAB18	**5’- TGCAGTACTGGTCATGTGGC-3’**
	**5’-GCAGTAGCATGAGTAGTGGGA-3’**

Nt LEA5	**5’- ATATGGCAATCCCATCCGCC-3’**
	**5’-GACGATGTTCCCCACCAAGT-3’**
